# Effects of Intermittent Energy Restriction Combined with a Mediterranean Diet on Reducing Visceral Adiposity: A Randomized Active Comparator Pilot Study

**DOI:** 10.3390/nu11061386

**Published:** 2019-06-20

**Authors:** Chloe E. Panizza, Unhee Lim, Kim M. Yonemori, Kevin D. Cassel, Lynne R. Wilkens, Michelle N. Harvie, Gertraud Maskarinec, Edward J. Delp, Johanna W. Lampe, John A. Shepherd, Loïc Le Marchand, Carol J. Boushey

**Affiliations:** 1 University of Hawaii Cancer Center, Honolulu, HI 96813, USA; CPanizza@cc.hawaii.edu (C.E.P.); ULim@cc.hawaii.edu (U.L.); KMurakam@cc.hawaii.edu (K.M.Y.); kevin@cc.hawaii.edu (K.D.C.); Lynne@cc.hawaii.edu (L.R.W.); Gertraud@cc.hawaii.edu (G.M.); johnshep@hawaii.edu (J.A.S.); 2 Prevent Breast Cancer Research Unit, Manchester University Hospital Foundation, National Health Service Trust, Wythenshawe, Manchester M23 9LT, UK; michelle.harvie@manchester.ac.uk; 3 School of Electrical and Computer Engineering, Purdue University, West Lafayette, IN 47907-2025, USA; ace@ecn.purdue.edu; 4 Public Health Sciences Division, Fred Hutchinson Cancer Research Center, Seattle, WA 98109, USA; jlampe@fredhutch.org

**Keywords:** DASH, East Asian American, intermittent energy restriction, Mediterranean diet, randomized trial, total adiposity, visceral adipose tissue

## Abstract

Intermittent energy restriction combined with a Mediterranean diet (IER+MED) has shown promise to reduce body fat and insulin resistance. In the Multiethnic Cohort Adiposity Phenotype Study, Japanese Americans had the highest visceral adipose tissue (VAT) when adjusting for total adiposity. We conducted this pilot study to demonstrate feasibility and explore efficacy of following IER+MED for 12 weeks to reduce VAT among East Asians in Hawaii. Sixty volunteers (aged 35–55, BMI 25–40 kg/m^2^, VAT ≥ 90 cm^2^ for men and ≥ 80 cm^2^ for women) were randomized to IER+MED (two consecutive days with 70% energy restriction and 5 days euenergetic MED) or an active comparator (euenergetic Dietary Approaches to Stop Hypertension (DASH) diet). Participants and clinic staff (except dietitians) were blinded to group assignments. IER+MED had significantly larger reductions in DXA-measured VAT and total fat mass (−22.6 ± 3.6 cm^2^ and −3.3 ± 0.4 kg, respectively) vs. DASH (−10.7 ± 3.5 cm^2^ and −1.6 ± 0.4 kg) (*p* = 0.02 and *p* = 0.005). However, after adjusting for total fat mass, change in VAT was not statistically different between groups; whereas, improvement in alanine transaminase remained significantly greater for IER+MED vs. DASH (−16.2 ± 3.8 U/L vs. −4.0 ± 3.6 U/L, respectively, *p* = 0.02). Attrition rate was 10%, and participants adhered well to study prescriptions with no reported major adverse effect. Results demonstrate IER+MED is acceptable, lowers visceral and total adiposity among East Asian Americans, and may improve liver function more effectively than a healthful diet pattern. ClinicalTrials.gov Identifier: NCT03639350.

## 1. Introduction

Excess adiposity contributes to an increased risk of cardiovascular disease, type 2 diabetes, and at least 13 cancers, including postmenopausal breast, endometrium, liver, gallbladder, pancreas, thyroid, kidney and colon cancer [1,2]. Visceral adiposity more so than subcutaneous adiposity is associated with the cardiovascular and metabolic consequences of obesity [3,4]. Higher visceral adipose tissue (VAT) levels result in increased circulating proinflammatory cytokines and adipokines, and a decrease in protective adipokines [4,5,6,7,8]. Also, non-alcoholic fatty liver disease (NAFLD) and steatohepatitis (NASH) have emerged as common liver diseases due to excess adiposity and are associated with type 2 diabetes, metabolic syndrome, and liver cancer [9].

Asians and Asian Americans are at a higher metabolic risk from excess adiposity compared to whites and other racial/ethnic populations [10]. In the Multiethnic Cohort Adiposity Phenotype Study (MEC-APS), conducted in Hawaii and California, we observed Japanese Americans preferentially store excess fat as VAT over subcutaneous adipose tissue (SAT) and also have higher levels of liver fat compared to participants of African American, Latino, Native Hawaiian or white ancestry after adjusting for total adiposity [11]. Consistent with this observation, among 23,830 men in the MEC over the median follow-up of 16.6 years, Japanese Americans showed a stronger association of higher body mass index (BMI) with the risk of hepatocellular carcinoma (HCC; relative risk (RR) for a 5 kg/m^2^ increase in BMI = 1.77) compared to African American, Latino, Native Hawaiian, and white men (RRs ranging 0.78–1.34) [12]. Other studies comparing Asians or Asian Americans to whites also found a higher prevalence of visceral obesity, NAFLD and metabolic syndrome [13,14], suggesting this group may benefit from an intervention aimed at reducing adiposity, especially VAT.

While energy restriction is the most common strategy for weight loss and visceral fat reduction [15], long-term adherence to continuous energy restriction (CER) is known to be difficult [16], and intermittent energy restriction (IER) has emerged as a promising alternative to CER [17]. IER includes periods of marked energy restriction (typically 60–75% below estimated energy requirements) on at least one day but no more than six days per week, interspersed with periods of normal or ad libitum energy intake [17,18,19,20,21]. In particular, Harvie et al. suggested combining two consecutive days of IER with five days of a Mediterranean (MED) type diet to promote satiety and high-quality nutrition [17,22]. The MED diet is primarily a plant-based diet rich in olive oil, olives, fruits, vegetables, whole grains, legumes and nuts with moderate amounts of dairy products (principally cheese and yogurt), fish, poultry, red wine, and limited amounts of red meat [23,24,25]. Adherence to a MED diet was also promoted for the management of NAFLD in joint clinical practice guidelines issued by the European Associations for the Study of Liver (EASL), Diabetes (EASD) and Obesity (EASO) [26]. These guidelines also recommended patients with NAFLD restrict energy intake, lose weight if overweight or obese, and incorporate aerobic exercise or resistance training [26]. Consistently, in the MEC-APS, we observed following a high-quality diet, e.g., a high MED index score, was inversely associated with adiposity, including VAT and liver fat as assessed by magnetic resonance imaging (MRI) [27]. Thus, adopting IER combined with a MED diet on the non-restricted days may help to reduce VAT, and assist with controlling other ectopic fat stores.

The primary aim of the present study was to finalize and implement a protocol for an intermittent energy restriction (IER) intervention to evaluate the effectiveness of a culturally adapted IER and MED combined diet (IER+MED) to reduce VAT among East Asian Americans. Secondary aims were to evaluate study retention and protocol adherence, and changes in total adiposity and metabolic risk biomarkers. East Asian women and men were also prioritized for this study as their traditional and acculturated East Asian diets are dissimilar compared to the IER+MED diet. Therefore, limiting enrollment to men and women of East Asian ancestry for participation in this pilot study allowed full attention to be directed to adapting their diets.

## 2. Materials and Methods

### 2.1. Study Design and Participants

The Healthy Diet and Lifestyle Study (HDLS) pilot was a two-arm randomized trial conducted between September 2016 and October 2017 at the University of Hawaii Cancer Center (UHCC) to demonstrate the feasibility of a nutritional intervention aimed at reducing visceral adiposity in East Asian middle-aged adults. This study included two clinic visits before the intervention (an eligibility visit and a baseline visit approximately one week apart (1.25 ± 1.2 weeks)), a 12 week intervention phase, a final clinic visit at Week 12, and a 6-month post-intervention telephone interview. Participants who responded to the study promotions, were first screened over the telephone to assess the inclusion criteria of East Asian ancestry (Japanese, Chinese, or Korean), residence in Honolulu County, body mass index (BMI) between 25 and 40 kg/m^2^, ages 35 to 55 years, and no serious health issues. Exclusion criteria included; smoking tobacco products or marijuana in the past two years, taking thyroid medication, prescription medication or insulin for type 1 or type 2 diabetes, anti-estrogen medication (women), anti-androgen medication (men), substantial change of weight of more than ± 10 kg in the past six months, following a special diet (e.g., vegan), or alcohol intake >15 drinks per week for men or >10 drinks per week for women. Ethnicity was self-reported and at least two biological grandparents of pure East Asian ancestry were required. Those screened as eligible were scheduled for an eligibility visit, which consisted of a fasting blood draw, anthropometric measurements, questionnaires (characteristics, medical history, medication list, physical activity), a whole-body dual energy X-ray absorptiometry (DXA) scan, and training participants how to complete a mobile food record^™^ (mFR^™^) [28,29,30,31] to capture images with their mobile device of foods/beverages before and after each eating occasion over 4 contiguous days. Final eligibility was determined based on general good health, normal blood count and biochemistry profile, and DXA-derived visceral fat area at L4-L5 intervertebral region ≥ 90 cm^2^ for men or ≥ 80 cm^2^ for women to target at-risk individuals for visceral obesity based on VAT distribution in the MEC-APS study [11]. The baseline clinic visit involved randomizing participants into the IER+MED group or the active comparator DASH group, education of diet and physical activity prescriptions, and reviewing information from mFRs^™^ collected between the eligibility and baseline visits. During the final clinic visit, measurements taken at baseline were repeated.

**Figure 1:**
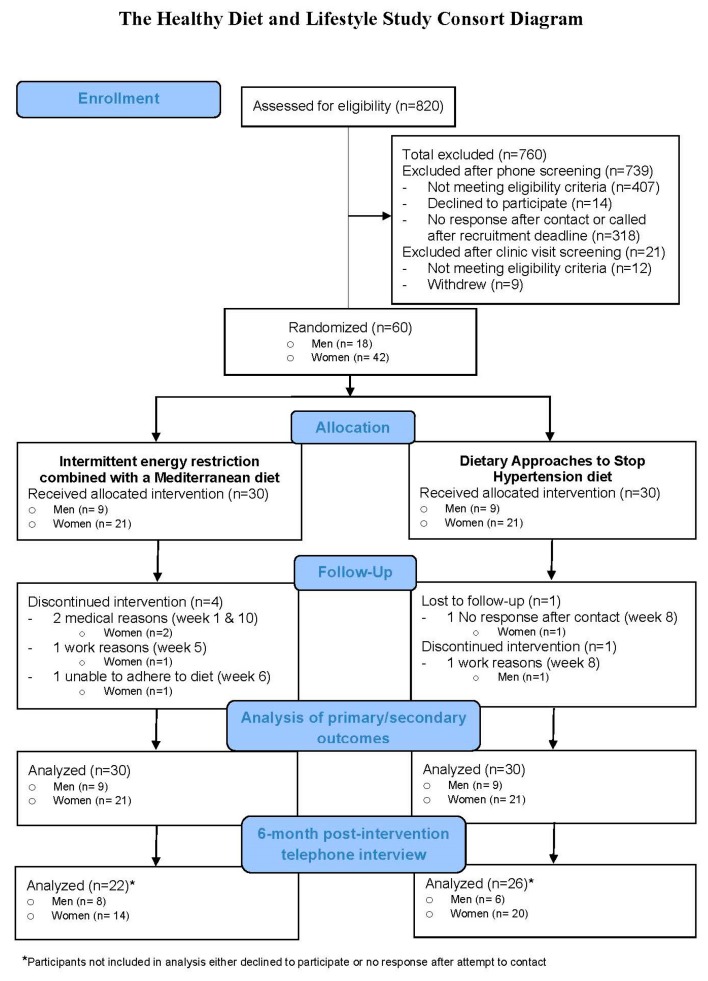
Consort diagram.

The enrollment goal was to recruit 70 persons to achieve a final sample of 50 persons to account for an attrition rate of ~23%, as reported in past studies [22]. Participants were recruited through advertisements in local newspapers, on radio stations, television news, social media, and email list-servs, and through distribution of brochures and flyers. A total of 820 people responded to the study promotions and of these, 760 people were excluded due to not meeting the eligibility criteria or declining to participate (Figure 1). As reimbursement for time and travel, each participant received a $50 gift card at the eligibility visit, $50 at the baseline clinic visit, and $50 at the final clinic visit, totaling $150 in gift cards to a state-wide supermarket chain. Participants were provided their whole-body DXA, BMI, and blood biochemistry panel results after the baseline and final clinic visits. All study procedures were approved by the University of Hawaii Institutional Review Board and written informed consent was obtained from all study participants.

### 2.2. Randomization and Masking

Randomization was implemented within strata defined by sex and VAT levels (above or below 150 cm^2^) and was blocked to ensure balance in assignment over the course of the study [32,33]. Stratified blocked randomization schedules were created by biostatisticians not involved in the intervention [33]. The assignments were printed and placed in opaque sealed envelopes with consecutive numbering and unique colors by strata. During the baseline clinic visit, research dietitians provided the participants with the next four consecutive envelopes in the appropriate stratum. The participant then selected and opened an envelope and shared the randomization information with the dietitian, who until then had been blinded to the group assignment. The participants (men, *n* = 18; women, *n* = 42) were randomized equally into either the intervention group or the DASH group. Recruitment and clinic staff were blinded to group assignments until after the 6-month post-intervention telephone interview. The current study was promoted as a healthy diet and lifestyle study, and study diets were identified as Diet 1 (for IER+MED) or Diet A (for DASH) to reduce any influence of familiarity with IER, MED or DASH diets.

### 2.3. Diet and Physical Activity Prescriptions

The intervention group was prescribed an IER+MED diet for 12 weeks. The IER component entailed a 70% energy restriction for two consecutive days with 34%, 33% and 33% of energy from protein, carbohydrate, and fat intakes, respectively. For the remaining five days per week, a euenergetic MED diet that met estimated energy requirements (EER), was prescribed with 25%, 45%, and 30% of energy from protein, carbohydrate, and fat, respectively. This regimen would achieve an overall energy restriction of 20% per week [22]. Participants self-selected which two consecutive days of the week to follow the IER protocol, and were asked to keep to those same two days throughout the study.

The active comparator group was prescribed a euenergetic DASH diet, which met EER, for 12 weeks, with 20%, 53%, and 30% of energy from protein, carbohydrate, and fat, respectively. An active comparator was used, instead of an inactive control, because all participants were at risk of poor metabolic conditions and would likely benefit from dietary support [34,35]. DASH was chosen as the comparator diet, as its regimen is broadly recognized as a healthful diet [36,37]. The DASH diet is rich in fruit, vegetables, low-fat dairy products, whole grains, and limits total fat, saturated fat and sodium [36,37]. All participants were advised to limit their alcohol intake, and the IER+MED group were restricted to zero alcoholic beverages on IER days. Increases in moderate to vigorous physical activity have been documented using accelerometers in past dietary interventions among intervention and control groups [38]. Therefore, to reduce confounding due to physical activity, we recommended both groups walk up to one hour per day, up to five days a week. The IER+MED group was encouraged to exercise on MED days only. EER for participants were determined based on their baseline body weight using the equations as published in the Dietary Reference Intakes (DRI) for energy for men and women 19 years and older [39]. The physical activity coefficient for men and women was assigned using the typical daily living activity descriptions from the DRI Calculator for Healthcare Professionals [40]. Information on hours in light, moderate and strenuous activity was estimated using the baseline physical activity questionnaire, which allowed dietitians to select an appropriate physical activity coefficient for each participant.

Both groups received an equal amount of planned dietitian guidance at baseline and during the intervention, although participants were encouraged to contact dietitians with any questions. The baseline face-to-face dietary consultation (45–60 minutes) with one of the three trial dietitians at the UHCC included instructions on how to follow their respective diet and physical activity plan. All participants received personalized diet booklets, individualized food lists and menus, and trackers to help them follow their plans at home. The IER+MED materials were originally developed and tested by Harvie et al. amongst white women in the UK [22,41]. Therefore, the food lists and menus were modified to provide examples of foods and beverages more commonly available in Hawaii, e.g., papaya, mango, brown rice, pak choi, sweet potato, tofu, edamame. All education materials were designed in 100 kcal increments, e.g., 1500 kcal, 1600 kcal, 1700 kcal. For example, participants with an EER of 2030 kcal were assigned an energy allotment rounded to the closest 100 kcal, i.e., 2000 kcal. If randomized to the IER+MED group, the energy allotment would have been 2000 kcal on MED days and 600 kcal on IER days. As an example, a food group prescription on MED days for the 2000 kcal plan was 8 carbohydrate servings, 8–14 protein servings, 7 fat servings, 3 dairy servings, 6 vegetable servings, and 4 fruit servings, and a maximum of three nutrient-poor treats of ≤ 150 kcal per week. On IER days, the primary restrictions were energy and carbohydrate, and a 600 kcal plan was comprised of 2–12 protein servings, 2 fat servings, 3 dairy servings, 5 vegetables servings and 1 fruit serving. If randomized to the DASH group, using the same example as above, the diet prescription for a 2000 kcal plan was 6–8 grain servings, 4–5 fruit servings, 2–3 dairy servings, 6 meat, poultry, or fish servings, 4–5 nuts, seeds, or legumes servings, 2–3 fats or oils servings, a maximum sodium intake of 2300 mg per day, and 5 or less sweets and added sugars servings per week. The personalized diet booklets detailed the amounts for servings of each food group and provided examples of types of foods to choose from within each food group. To support the dietary counseling, study dietitians underwent training in behavioral change strategies using the Body and Soul program [42,43,44]. In particular, the training focused on motivational interviewing techniques, ensuring the dietitians practiced reflective listening and provided positive affirmations rather than relying heavily on persuasion or advice giving [42,43,44]. Dietitians contacted participants at Weeks 1, 2, 3, 4, 6, 8 and 10, primarily by telephone, and met in person with participants during the final clinic visit, to assess participants’ compliance to the intervention plans and guide positive behavioral change.

### 2.4. Study Measurements

Dietary intakes were assessed using the mFR^™^ completed at baseline, between Weeks 5–6, and at Week 11. The mFR^™^ is designed to capture images of foods/beverages before and after each eating occasion and allows for automatic uploading of images to a secure cloud-based server when in 3G/4G/Wi-Fi range [28,29,30,31]. During the eligibility visit, the mFR^™^ app was loaded onto each participant’s mobile device by a study dietitian. Participants were trained on how to use the mFR^™^ and provided with a fiducial marker (a small reference device of known dimensions and colors) to include in images [30,31]. All participants were asked to use the mFR^™^ over four contiguous days including at least one weekend day to capture a baseline mFR^™^ between the eligibility and baseline clinic visits. After the baseline clinic visit, participants in the IER+MED group were asked to keep mFRs^™^ of their two IER days bookended by two MED days, e.g., MED-IER-IER-MED at Weeks 5–6 and Week 11. Participants in the DASH group were asked to keep to the same recording days as their baseline record for recording their Weeks 5–6 and Week 11 mFRs^™^. Images from the mFR^™^ were reviewed in person with a dietitian at the baseline and the final clinic visits, and over the phone at Week 6. All participants were willing to download the app, except five could not due to owning an incompatible phone (*n* = 3), full phone memory (*n* = 1), or phone lacking features to run app (*n* = 1). Consequently, these five participants completed written records [45,46]. Data entry of before and after images of food and beverages followed the methods by Kerr et al [29,47]. Briefly, dietitians underwent analyst training before entering the food and beverage data into RapidCalc, a dietary data entry program developed at UHCC [48,49]. Training consisted of identifying the amount and type of food in test mFR^™^ images. This task was completed with the assistance of a fiducial marker [50] for size estimation, and an additional foods questionnaire (completed by participants at baseline) to help identify occluded foods, e.g., type of milk in tea or coffee. A priori, only dietary records with at least two days of recording and at least one eating occasion captured on each day were to be included in the analysis.

Clinic measures were taken at baseline and Week 12. Anthropometric, body composition, fasting bloods, and blood pressure measures were collected using the same protocol as Lim et al [11]. Briefly, whole-body composition was determined by DXA (Hologic Discovery A fan-beam densitometer, Hologic Inc. (Bedford, MA, USA) using APEX 3.3). Fat mass and lean mass were estimated for the whole body, trunk, arms, and legs, from which skeletal muscle mass was derived [51,52,53]. For VAT and SAT outcomes, we used visceral and subcutaneous fat area estimates for L4-L5 derived from DXA parameters. Trained technicians obtained measurements of height, weight, and circumferences of the waist and hip. Fasting blood samples were processed at the UHCC and analyzed at the UHCC Analytical Biochemistry Shared Resource Laboratory for plasma levels of total cholesterol, high-density (HDL and low-density (LDL) cholesterol, glucose, insulin, alanine transaminase (ALT), and aspartame transaminase (AST). Blood pressure in the left arm was measured in a sitting position after 20 minutes of rest using a digital monitor (Omron HEM-907XL, Omron Healthcare, Inc. (Lake Forest, IL, USA)). Physical activity levels (PALs) were assessed at baseline using a physical activity questionnaire previously validated for the MEC [54]. The questionnaire was designed to reflect average physical activity per day completed in the preceding year, including moderate-to-vigorous activity [54]. The baseline physical activity questionnaire was modified for the Week 12 visit, with participants being asked to recall physical activity in the preceding week.

During the telephone calls at Weeks 1, 2, 3, 4, 6, 8 and 10, and the in person visit at Week 12, participants in the IER+MED group self-reported how many IER days they had successfully completed in the most recent week, i.e., 0, 1, or 2 days. All participants were also asked, “How well have you been following your diet plan? On a scale of zero to ten with zero being not at all, four being somewhat, and ten being following the plan very well, where would you place yourself?” and “How well have you been following your physical activity plan? On a scale of zero to ten with zero being not at all, four being somewhat, and ten being following the plan very well, where would you place yourself?”. Participants were also encouraged to report any minor or major adverse effects experienced during the study (e.g., adverse reactions associated with performing DXA, phlebotomy, or from following the diet).

### 2.5. 6-month Post-Intervention Telephone Interview

At 6 months post-intervention, participants who completed the study were interviewed over the telephone. These calls were conducted by trained recruitment staff not involved in the 12-week intervention counseling. Quantitative questions included current weight, still following the intervention diet plan (yes/no) and extent (same, better, not as well), willingness to follow the prescribed diet longer than 3 months (yes/no), and interest in nutrition/food preparation classes (yes/no). Open-ended qualitative questions related to current health issues, physical activity, description of type of diet currently being followed, and suggestions on how the study could be improved were also asked. This paper summarizes and reports only the responses to the quantitative questions.

### 2.6. Statistical Analysis

Continuous variables are reported as means ± SDs or SEMs, and categorical variables are reported as counts and percentages. The analysis followed an intention-to-treat-analyses, where all individuals were analyzed in a randomization group, regardless of compliance. A linear mixed model was fit for each outcome. This model uses of all available data to estimate the treatment effects over time using maximum likelihood estimation under a missing-at-random assumption [55,56]. The model included an indicator variable for intervention group (IER+MED vs. DASH), indicator variable for time (Week 12 vs. baseline, or Weeks 5–6 vs. baseline and Week 11 vs. baseline for diet), and interaction terms between group and time. The F test was used to assess the intervention effect, defined as the contrast of change in IER+MED minus change in DASH. Outcome variables included: dietary intakes, body measurements, biomarkers, and physical activity. The following comparisons were made across time points: Week 12 vs. baseline for body measurements, physical activity, and biomarkers, and Weeks 5–6 vs. baseline, Week 11 vs. baseline, and Weeks 5–6 vs. Week 11 for diet. No transformations of the outcomes were needed to meet model requirements as values for change over time were approximately normal and homoscedastic. To test the specific effects of the IER+MED intervention on VAT and biomarkers independent of those on total adiposity, additional models were run adjusting for concurrent total fat mass. Model-predicted adjusted means at each time point for each group were computed. Diet data are represented as group mean daily intakes. For DASH, the mean across four food record days were taken, while for the IER+MED group, means were computed by IER and MED days and then an overall average was computed, weighting the IER mean by 2 and the MED mean by 5. Per protocol analyses, changes in diet and body measurements were also conducted only among those completing the intervention (i.e., had a Week 12 assessment). For per protocol analyses, diet data were also reported separately for IER and MED days. To verify the reported change in energy intake over time, for both study groups, the expected weight change at 12 weeks was compared to measured weight change at Week 12. An energy deficit of 500 to 1000 kcal/day is estimated to result in a weight loss of 0.45 to 0.90 kg/week [57,58]. The average difference per day between energy intakes at baseline and Week 11 was estimated as the average of the change from baseline to Weeks 5–6 and the change from Weeks 5–6 to Week 11, which were calculated from a mixed model of energy on time, for both groups. The average change in weight in pounds was then calculated as the estimated change in energy per day from baseline to Week 11 converted to pounds of weight loss as [change in energy/500] · 12 weeks. A 95% confidence interval for change in weight was computed by converting the limits of a 95% confidence interval for change in energy.

Alcohol, vitamin and mineral intake, and the proportion of participants meeting the US estimated average requirement (EAR) for vitamin and mineral intake at baseline, Weeks 5–6 and 11 are reported for both groups. Statistical significance was defined as *p* < 0.05. Data were analyzed using SAS version 9.4 software (SAS Institute Inc., Cary, NC, USA) and IBM SPSS Statistics version 25 software (IBM Corp., Armonk, NY, USA).

## 3. Results

### 3.1. Study Population

The stratified randomized sampling was successful, with men and women being distributed evenly between groups, and participants with high (<150 cm^2^) and very high (≥150 cm^2^) VAT being distributed almost equally between groups (Table 1). After 12 weeks, four participants dropped out of the IER+MED group and two out of the DASH group; therefore, 87% (*n* = 26) of participants in the IER+MED group and 93% (*n* = 28) in the DASH group completed the study (Figure 1). One participant from the IER+MED group dropped out due to not being able to adhere to the diet and the other five participants dropped out for reasons unrelated to the study (e.g., work, medical reasons). There were no major adverse effects reported during the intervention.

### 3.2. Intervention Adherence

The IER+MED participants completed 90.6% of the allocated IER days. Ninety six percent of these IER days were completed as two consecutive IER days and the remainder completed at least one IER day per week. For the IER+MED group, the mean self-rated compliance to the diet prescription and the self-rated compliance to the physical activity prescription were 7.7 at Week 1, Week 6, and Week 12 using the scale of 0–10. For the DASH group, the respective self-rated compliance rates were 6.7, 6.8, and 6.4 for diet and 6.4, 7.3, and 6.9 for physical activity. Data from the physical activity questionnaires support there being no significant change in physical activity from baseline to Week 12 for within the IER+MED group (1.58 ± 0.22 to 1.43 ± 0.23 hours of moderate or vigorous activity/day, respectively, *p* = 0.49) and the DASH group (1.39 ± 0.22 to 1.27 ± 0.22 hours of moderate or vigorous activity/day, *p* = 0.58) or between groups (*p* = 0.91). Both groups met the physical activity recommendations at the beginning and end of the intervention.

Overall, there was a 23% decrease in mean daily energy intake for the IER+MED group from baseline (1590 kcal) to Weeks 5–6 (1227 kcal), and a 28% decrease between baseline and Week 11 (1152 kcal) (Table 2). Mean energy allotment for the IER days was 692 kcal (range 540 to 960 kcal). Estimated mean energy intakes on IER days were 960 kcal at Weeks 5–6 and 929 kcal at Week 11 (Table S1). For the MED days, the mean energy allotment was 2307 kcal (range 1800 to 3200 kcal). Estimated mean energy intakes on MED days were 1222 kcal at Week 5–6 and 1144 kcal at Week 11. For the DASH group, energy intake decreased by 22% between baseline (1803 kcal) and Weeks 5–6 (1414 kcal) and by 16% between baseline and Week 11 (1507 kcal) as seen in Table 2. Mean energy allotment was 2300 kcal (range 1800 to 3400 kcal).

The prescriptions for percentage energy from protein, carbohydrates and total fats for the IER+MED group were approximately 28%, 42%, and 31%, respectively (weighted for two IER days and five MED days). By Week 11, participants in this group increased their percentage energy from protein from 18.7% to 25.7%, almost matching the prescription of 28%. The mean percentage energy from carbohydrates decreased from 44.8% to 35.1% which was lower than the goal of 42%. Percentage energy from total fats increased from 36.6% to 40.2%, as compared to the recommended of 31%. When examined as absolute intake, participants in the IER+MED decreased their mean intakes of total fats from 64.7 g at baseline to 51.8 g at Week 11, and their mean intakes of carbohydrates from 180 g to 103 g. Since the drop in grams of carbohydrates was proportionally larger than the drop in total fats, percentage of energy from carbohydrates decreased and percentage energy from total fats increased. For the DASH group the recommended percentage energy from protein, carbohydrates and total fats was 20%, 53%, and 30%, respectively. By Week 11, the DASH group increased percentage energy from protein from 17.3% to 18.9%, with the goal of reaching 20%. At baseline, percentage energy from carbohydrates and total fats were 44.1% and 38.3%, respectively, and did not significantly change by Week 11. Assessing absolute intakes, mean carbohydrate intakes dropped from 198 g to 169 g and mean intakes of total fats decreased from 77.6 g to 62.7 g. Total energy intake also decreased by Week 11; therefore, the drop in grams of carbohydrates and total fats consumed did not affect percentage of energy from these macronutrients. For both the IER+MED and DASH groups there were no significant changes in total energy (kcal); percentage energy from total protein, fat, or carbohydrate; and grams of protein, fat, or carbohydrate consumed between Weeks 5–6 and Week 11. In per protocol analyses, similar patterns were seen (Table S1).

At Week 11, and only among the IER+MED group, reductions in calcium, thiamin, and folate intakes were observed, as well as, the proportion of participants meeting the EAR for calcium, thiamin, and folate (Tables S2–S3). A more detailed description of alcohol and micronutrient intake and proportion of participants meeting the EAR for micronutrients can be found in the supplementary tables (Tables S2–S3).

### 3.3. Changes in Anthropometric, DXA, and Biomarker Measurements at Week 12

Both the IER+MED and the DASH groups experienced significant reductions in all anthropometric and DXA measurements from baseline to Week 12 (Table 3). Between groups, the IER+MED group had a significantly greater loss of weight, BMI, waist circumference, hip circumference, percentage body fat, fat mass, muscle mass, total lean body mass, VAT, and SAT compared to the DASH group (Table 3). These decreases in anthropometric and DXA measures were close to double for the IER+MED group compared to the DASH group and over three times the amount for change in SAT. In the IER+MED group, approximately 73% of participants lost 5% or greater of their weight and 27% of participants lost 10% or greater of their weight. In the DASH group, these were 32% and 7%, respectively. Per protocol analyses, showed similar results for change in anthropometric and DXA measurements between and within groups (Table S4). After adjusting for concurrent total fat mass, change in VAT was no longer significantly different between groups (IER+MED −8.6 ± 3.1 cm^2^ vs. DASH −3.7 ± 2.6 cm^2^, *p* = 0.188). The VAT/SAT ratio did not change significantly for either group between baseline and Week 12 (Table 3). Based on the average difference per day between energy intakes at baseline and Week 11, and the guidelines of an energy deficit of 500 to 1,000 kcal/day is estimated to result in a weight loss of 0.45 to 0.90 /week, the expected mean (95% CI) weight loss for the IER+MED group at Week 12 was 5.1 (2.5–7.8) kg. Actual mean weight loss at Week 12 was 5.9 kg; therefore, fell within the expected range. For the DASH group, expected mean weight loss at Week 12 was 3.6 (0.2–6.9) kg. Actual weight loss was 3.3 kg; therefore, also fell within the expected range. All fasting blood biomarkers (total and LDL cholesterol, triglycerides, insulin, ALT, and AST), except HDL cholesterol, and systolic and diastolic blood pressure significantly improved in the IER+MED group, whereas only triglycerides, insulin, and blood pressure improved in DASH (Table 4). Only the improvement in ALT was significantly greater in the IER+MED group compared to the DASH group (*p* = 0.04), which was maintained after adjusting for concurrent total fat mass (−16.2 ± 3.8 U/L vs. −4.0 ± 3.6 U/L, respectively) (*p* = 0.02).

### 3.4. 6-month Post-Intervention Telephone Interview

Among participants completing the 6-month post-intervention telephone interview, for the IER+MED group there was no significant change in body weights measured at Week 12 and self-reported at 6 months post study (Table 5). For the DASH group there was a significant increase in body weights (*p* = 0.03). During the post-intervention telephone call, both the IER+MED and DASH groups reported physical activity of over 3.5 hours/week (mean). In addition, 71.4% of participants in the IER+MED group, compared to 88.0% in the DASH group, reported they were able to follow the prescribed diet longer than three months after the study finished (Table 5). The mean amount of time participants could follow the diet prescription was 5.0 months for the IER+MED group vs. 3.6 months for the DASH group. In addition, almost two-thirds, 66.7%, of participants in the IER+MED group were still following their diet prescription at the time of the post-intervention telephone call, with 6.7% reporting they were still following the diet protocol the same as they were when completing the study. For the DASH group, 44.0% of participants were still following their diet prescription at the time of the post-intervention telephone call and 25.0% reported they were still following the diet protocol the same as during the study.

## 4. Discussion

This is the first known pilot study to evaluate the effectiveness of a culturally adapted IER+MED vs. an active comparator (DASH diet) to reduce DXA measured VAT among East Asian Americans. This study was also unique in that the mFR was used to capture dietary intake. Despite the IER+MED diet being different to traditional and acculturated Asian diets, participants complied well to prescriptions. For both study arms, the recommendations, which appeared most difficult to achieve were those for carbohydrate and fat, with percentage of energy from carbohydrates being consistently lower than recommended and percentage of energy from total fats being consistently higher. Despite reductions in energy intake, the proportion of fiber in the diet, for the IER+MED and DASH groups, was higher at Week 12 than baseline. Both study groups had significant reductions in VAT, weight, and total adiposity. Although loss of VAT was greater in IER+MED than in DASH, this appeared to be due to the greater loss of total fat in IER+MED as there was no significant group difference after adjusting for concurrent total fat mass. For IER+MED, we observed general improvements in metabolic risk biomarkers, particularly in total and LDL cholesterol, triglycerides, ALT, AST, insulin, systolic and diastolic blood pressure. Only the reduction in ALT was significantly greater in IER+MED vs. DASH, a difference maintained after adjusting for concurrent total fat mass. This indicates a potentially greater benefit of IER+MED on liver function, compared to a healthful dietary pattern [59]. The observed results were not due to differences in physical activity as both groups reported similar levels of physical activity at the beginning and end of the intervention, and these levels met the physical activity recommedations. This pilot study demonstrated that an IER+MED vs. DASH intervention can be successfully conducted with East Asian Americans, and with a low attrition rate of 10%.

Consistent with our results, other IER trials involving two energy restricted days as consecutive or non-consecutive restricted days (termed “5:2” [55]) have reported high study retention and protocol adherence [22,55,60,61]. Attrition rates range from 4.2% to 23% [22,55,60,61], which supports observations of participants being able and willing to complete “5:2” trials. In the “5:2” study by Harvie et al, a study arm was prescribed a 70% energy restriction for two consecutive days and 5 non-restricted days following a euenergetic MED diet [22]. Over three months, participants in the IER study arms in the Harvie et al trial completed 74–76% of their two IER days, indicating high study compliance [22]. Similarities between the current study and the Harvie et al. trials were energy intake was higher than prescribed on IER days, and energy and carbohydrate intakes were lower than prescribed on non-restricted days [22]. This highlights that participants do well at completing their IER days, but may need extra support with meeting their energy and carbohydrate prescriptions. Results from the current study also support previous findings that participants do not over eat on non-restricted days [18,22]. Harvie et al [22] hypothesized that the reduced intake on non-restricted days could be due to behavioral aspects of following IER (e.g., IER made participants more aware of habitual intakes, and increased awareness of appetite and hunger). Also, for both groups, dietitians promoted consuming high quality unprocessed diets on euenergetic days, which may have resulted in participants consuming less energy than prescribed [62]. As part of the post-intervention follow up call, the majority of participants from both groups reported nutrition classes and/or food preparation classes would have been helpful. Incorporating nutrition classes and/or food preparation classes may be a useful strategy to improve compliance to dietary prescriptions [63].

We observed no preferential loss of VAT in the IER+MED group compared to the DASH group after adjusting for concurrent total fat mass. The results of this analysis could be clinically true if the DASH group was able to lose similar amounts of fat mass to the IER+MED group and maintain this loss. However, results suggest that IER+MED is easier to follow than the euenergetic DASH, and weight loss easier to maintain. For example, self-rated dietary compliance scores were higher for IER+MED (7.7 at Weeks 1, 6 and 12) compared to DASH (6.7, 6.8, and 6.4, respectively), and at 6-months post-intervention 66.7%, of the IER+MED group vs. 44.0% of the DASH group reported still following their diet prescriptions. In addition, between the Week 12 visit and the 6-month post-intervention telephone interview, amongst participants completing the call, self-reported weight suggested that weight loss was maintained in the IER+MED group and that weight increased in the DASH group. These results are promising as CER is known to be difficult to follow long term [16]; therefore, IER may be an easier alternative to adopt.

VAT data were not collected at 6-months post intervention; however, given the self-report of weight maintenance, it is likely that loss of VAT was also maintained in the IER+MED group [15]. The only known “5:2” IER trial to assess the effect of IER on VAT is the HELENA trial carried out over 50 weeks [55]. This trial found that IER did not exert stronger effects on VAT loss than CER [55]; however, their trial incorporated two non-consecutive restriction days. Incorporating two consecutive vs. non-consecutive restriction days may have differing effects on health outcomes. The former may likely produce higher reductions on insulin resistance and percentage body fat [22,55]. Therefore, further long term studies are needed to assess the effects of “5:2” trials with 2 consecutive restriction days on VAT. The IER+MED and DASH groups reduced VAT proportionally to their change in total adiposity and the difference between groups may be due to the difference in energy intakes. Diets limited in sodium and more in line with the DASH diet, have been associated with lower VAT [27,64], which likely contributed, along with the observed decrease in energy intake, to the loss of VAT in the DASH group. These improvements in VAT may explain the lack of significant difference between groups in most metabolic risk biomarkers. The greater improvement in ALT in the IER+MED group vs. the DASH group is important as ALT is a biomarker for NAFLD [59]. Significant reductions in ALT in the IER+MED group even after adjusting for change in total adiposity suggest improvements in liver function, beyond that obtained by VAT reduction alone [59]. However, liver fat measurement e.g., by abdominal MRI scans are needed to confirm this. The decrease in ALT when following IER+MED is consistent with the European guidelines for NAFLD, which recommend following a MED diet and energy restriction to improve NAFLD [26].

For the IER+MED group, there was a reduction in dietary calcium, thiamin, and folate intakes, and in the proportion of participants meeting the EAR for calcium, thiamin, and folate. Past “5:2” studies have also reported reductions in micronutrients [22,61]; for example, Harvie et al expressed concern about lower intakes of calcium, iron, zinc, vitamins A and D and fiber in the IER groups [22]. Previous “5:2” trials, have prescribed a healthful diet [22,55] or recommended participants follow their usual diet [60,61] on non-restricted days. Given the large energy deficit on restricted days, prescribing a balanced diet on non-restriction days along with a nutritional supplement may help to limit any possible deficiencies. In the current study, blood measurements to assess nutritional deficiencies were not completed. Across “5:2” trials, including the current study, there were no reported serious adverse effects [22,55,61].

The strengths of the design for the current study include the stratified random design, the inclusion of an active comparator, blinding of participants and study staff (other than dietitians) to group allocations, the ethnic/racial tailoring of the intervention prescriptions, and providing the same physical activity recommendations for both study arms. Another strength was the low attrition rate, which added to the validity and reliability of study results. The reliability is evident from the similarity in results between the intention-to-treat analyses using all participants (*n* = 60) and the per protocol analyses using only participants who completed the study (*n* = 54). Additional strengths were the use of the mFR^™^ for assessing dietary intake, which was used to help generate responses to study promotions, encourage adherence during the intervention, and allowed the intervention dietitians to monitor dietary intakes in real time [28,29,30,65], and the novel use of DXA to measure VAT.

Limitations of the current pilot study include the small sample size, which may have limited statistical power to show small differences between groups. Also, the possible misreporting of dietary intake, with underreporting of dietary intakes being common among people with overweight or obesity [66,67]. However, weight loss achieved by participants in the IER+MED and DASH groups corresponded well to the average change in daily energy intake between baseline and Week 11, indicating at the group level, the dietary data was collected and analyzed accurately [57,58]. Another limitation is that results from the 6-month post-intervention telephone interview may not be representative of the study sample due to not all participants responding and the self-reported nature of the information collected. There have been limited studies comparing DXA-based VAT measures with CT or MRI and, in the few studies reported, the DXA results overestimated VAT, particularly in individuals with higher VAT levels [68,69,70].

## 5. Conclusions

In summary, this randomized pilot study, testing the effects of IER+MED vs. an active comparator DASH diet on VAT levels, was successfully conducted among a relatively small sample of East Asian Americans in Hawaii. Despite the prior belief among the investigators that the IER+MED diet would be challenging to adopt among East Asian Americans, the participants complied well to the culturally adapted study prescriptions and the attrition rate was low. Visceral adiposity, as well as total fat mass and ALT, were reduced to a greater extent in the IER+MED diet group than in the DASH diet group, possibly because the observed decrease in energy intake was greater in the IER+MED group. Within groups, changes in VAT may have resulted from changes in macronutrient intakes. IER+MED was superior to DASH in improving ALT since this improvement was not explained by the greater reduction in total fat mass. The results of this pilot study are promising, and further studies addressing a larger sample of men and women are needed to confirm the effectiveness of the IER+MED diet on change in VAT, and its possible beneficial effect on liver fat, in the short term and long term.

## Figures and Tables

**Table 1:** Randomization and baseline characteristics of study participants by the intermittent energy restriction combined with a Mediterranean diet (IER+MED) and dietary approaches to stop hypertension (DASH) groups.

Variable	Randomization Group	
	IER+MED (*n* = 30)	DASH (*n* = 30)
Men	9	9
Visceral adipose tissue category (%)		
High (90 < 150 cm^2^)	6 (67%)	6 (67%)
Very high (≥ 150 cm^2^)	3 (33%)	3 (33%)
Women	21	21
Visceral adipose tissue category (%)		
High (80 < 150 cm^2^)	13 (62%)	14 (67%)
Very high (≥ 150 cm^2^)	8 (38)	7 (33%)
Characteristics (Men and Women)		
Age (years)	48.4 ± 4.7	46.2 ± 5.4
Height (m)	1.6 ± 0.1	1.6 ± 0.1
Weight (kg)	79.3 ± 12.5	81.0 ± 12.5
Visceral adipose tissue area (cm^2^)	134.6 ± 6.4	135.3 ± 6.4
Body mass index (kg/m^2^)	30.5 ± 3.5	30.8 ± 3.3
Moderate or vigorous physical activity (hours/day)	1.6 ± 0.2	1.4 ± 0.3
Ethnicity (%)		
Chinese	23.3	6.7
Japanese	56.7	63.3
Korean	10.0	13.3
Mixed Asian	10.0	16.7

Data are presented as mean ± standard deviation (SD) or number (%). IER+MED: Intermittent energy restriction combined with a Mediterranean diet. DASH: Dietary Approaches to Stop Hypertension diet.

**Table 2:** Dietary intake assessed using 4-day mobile food records (mFR™) captured by participants in the IER+MED group (*n* = 30) and the DASH group (*n* = 30) across three time points ^1^.

Variable	Baseline	Weeks 5–6	*p* ^2^	Week 11	*p* ^3^
Energy (kcal)					
IER+MED ^4^	1590 ± 078	1227 ± 085	<0.0001	1155 ± 077	<0.0001
DASH	1803 ± 111	1414 ± 096	0.001	1507 ± 100	0.001
Protein (g)					
IER+MED	73.0 ± 3.6	74.9 ± 5.0	0.692	72.7 ± 4.2	0.946
DASH	76.5 ± 4.6	66.1 ± 4.8	0.055	70.6 ± 4.7	0.128
Protein (% energy)					
IER+MED	18.7 ± 0.7	24.9 ± 1.0	<0.0001	25.7 ± 1.1	<0.0001
DASH	17.3 ± 0.6	18.7 ± 0.5	0.115	18.9 ± 0.5	0.011
Carbohydrate (g)					
IER+MED	180 ± 11	115 ± 10	<0.0001	103 ± 09	<0.0001
DASH	198 ± 13	160 ± 12	0.013	169 ± 12	0.008
Carbohydrate (% energy)					
IER+MED	44.8 ± 1.5	37.3 ± 1.6	<0.0001	35.1 ± 1.6	<0.0001
DASH	44.1 ± 1.2	44.8 ± 1.1	0.650	45.2 ± 1.3	0.418
Total fat (g)					
IER+MED	65 ± 4	53 ± 5	0.015	52 ± 4	0.001
DASH	78 ± 6	57 ± 4	<0.0001	63 ± 5	0.001
Total fat (% energy)					
IER+MED	36.6 ± 1.1	39.1 ± 1.4	0.140	40.2 ± 1.2	0.014
DASH	38.3 ± 1.1	36.7 ± 1.1	0.183	36.8 ± 1.3	0.234
Saturated fatty acids (% energy)					
IER+MED	11.5 ± 0.4	11.1 ± 0.6	0.560	11.6 ± 0.6	0.854
DASH	11.7 ± 0.5	11.0 ± 0.4	0.092	11.8 ± 0.5	0.830
Monounsaturated fatty acids (% energy)					
IER+MED	13.6 ± 0.4	15.0 ± 0.6	0.055	15.9 ± 0.6	0.001
DASH	14.5 ± 0.5	13.6 ± 0.5	0.111	13.9 ± 0.6	0.357
Polyunsaturated fatty acids (% energy)					
IER+MED	8.4 ± 0.5	9.6 ± 0.5	0.093	8.8 ± 0.5	0.426
DASH	9.0 ± 0.5	9.0 ± 0.6	0.994	7.7 ± 0.4	0.093
Dietary fiber (g)					
IER+MED	13.0 ± 1.0	12.8 ± 0.9	0.874	11.9 ± 1.0	0.297
DASH	13.4 ± 1.1	14.1 ± 1.3	0.661	13.7 ± 1.0	0.814

Data are presented as mean/day ± standard error of the mean (SEM). IER+MED: Intermittent energy restriction combined with a Mediterranean diet. DASH: Dietary Approaches to Stop Hypertension diet.^1^ All data analyzed using an intention-to-treat approach with a linear mixed model for all 60 participants. ^2^ Within group difference from baseline to Weeks 5–6. ^3^ Within group difference from baseline to Week 11. ^4^ Weighted for five Mediterranean diet (MED) days and two intermittent energy restriction (IER) days.

**Table 3:** Baseline, Week 12, and change in anthropometric measures within and between the IER+MED group (*n* = 30) and DASH group (*n* = 30) ^1^.

Variable	Baseline	Week 12	*p* ^2^	Change	*p* ^3^
Weight (kg)					
IER+MED	79.3 ± 2.2	73.4 ± 2.2	<0.0001	−5.9 ± 0.7	0.007
DASH	81.0 ± 2.2	77.8 ± 2.2	<0.0001	−3.3 ± 0.6	
Body mass index (kg/m^2^)					
IER+MED	30.5 ± 0.6	28.3 ± 0.6	<0.0001	−2.2 ± 0.2	0.002
DASH	30.8 ± 0.6	29.6 ± 0.6	<0.0001	−1.2 ± 0.2	
Waist circumference (cm)					
IER+MED	100.3 ± 1.6	93.3 ± 1.6	<0.0001	−6.9 ± 0.8	0.026
DASH	100.7 ± 1.6	96.2 ± 1.6	<0.0001	−4.5 ± 0.7	
Hip circumference (cm)					
IER+MED	107.7 ± 1.3	102.5 ± 1.3	<0.0001	−5.3 ± 0.5	0.021
DASH	107.3 ± 1.3	103.9 ± 1.3	<0.0001	−3.4 ± 0.5	
Body fat (%)					
IER+MED	33.4 ± 1.2	31.3 ± 1.2	<0.0001	−2.0 ± 0.4	0.021
DASH	33.0 ± 1.2	32.1 ± 1.2	0.023	−0.8 ± 0.4	
Fat mass (kg)					
IER+MED	26.4 ± 1.1	23.1 ± 1.1	<0.0001	−3.3 ± 0.4	0.005
DASH	26.4 ± 1.1	24.9 ± 1.1	<0.0001	−1.6 ± 0.4	
Muscle mass (kg)					
IER+MED	21.9 ± 0.9	20.8 ± 0.9	<0.0001	−1.1 ± 0.2	0.013
DASH	22.3 ± 0.9	21.8 ± 0.9	0.005	−0.5 ± 0.2	
Total lean body mass (kg)					
IER+MED	52.6 ± 1.8	50.4 ± 1.8	<0.0001	−2.3 ± 0.4	0.040
DASH	54.3 ± 1.8	53.1 ± 1.8	0.002	−1.2 ± 0.4	
Visceral adipose tissue area (cm^2^)					
IER+MED	134.6 ± 6.4	112.0 ± 6.5	<0.0001	−22.6 ± 3.6	0.022
DASH	135.3 ± 6.4	124.5 ± 6.5	0.003	−10.7 ± 3.5	
Subcutaneous adipose tissue area (cm^2^)					
IER+MED	373.1 ± 16.2	324.9 ± 16.4	<0.0001	−48.2 ± 6.4	<0.0001
DASH	359.0 ± 16.2	344.0 ± 16.3	0.018	−15.0 ± 6.1	
VAT/SAT ratio ^4^					
IER+MED	0.38 ± 0.02	0.36 ± 0.02	0.157	−0.01 ± 0.01	0.825
DASH	0.39 ± 0.02	0.37 ± 0.02	0.076	−0.01 ± 0.01	

Data are presented as mean ± standard error of the mean (SEM). IER+MED: Intermittent energy restriction combined with a Mediterranean diet. DASH: Dietary Approaches to Stop Hypertension diet. ^1^ All data analyzed using an intention-to-treat approach with a linear mixed model for all 60 participants. ^2^ Within group difference from baseline to Week 12. ^3^ Between group difference (IER+MED vs. DASH) from baseline to Week 12. ^4^ Ratio of visceral adipose tissue area to subcutaneous adipose tissue area.

**Table 4:** Baseline, Week 12, and change in metabolic risk biomarkers within and between the IER+MED group (*n* = 30) and the DASH group (*n* = 30) ^1^.

Variable	Baseline	Week 12	*p* ^2^	Change	*p* ^3^
Cholesterol (mg/dL)					
IER+MED	237.0 ± 10.3	219.5 ± 10.3	0.009	−17.4 ± 6.4	0.356
DASH	250.0 ± 10.0	240.9 ± 10.0	0.149	−9.1 ± 6.2	
HDL cholesterol (mg/dL)					
IER+MED	38.1 ± 2.4	39.6 ± 2.4	0.396	1.5 ± 1.8	0.610
DASH	32.1 ± 2.3	34.9 ± 2.3	0.110	2.8 ± 1.7	
LDL cholesterol (mg/dL)					
IER+MED	178.5 ± 9.2	164.5 ± 9.2	0.019	−14.0 ± 5.8	0.585
DASH	188.6 ± 9.1	179.1 ± 9.1	0.104	−9.5 ± 5.8	
Triglycerides (mg/dL)					
IER+MED	101.9 ± 26.2	77.1 ± 26.2	0.004	−24.8 ± 8.2	0.809
DASH	165.5 ± 25.2	143.5 ± 25.2	0.008	−22.0 ± 7.9	
Alanine transaminase (U/L)					
IER+MED	33.8 ± 3.2	20.1 ± 3.2	0.001	−13.8 ± 3.7	0.038
DASH	19.5 ± 3.1	16.6 ± 3.1	0.419	−2.9 ± 3.6	
Glucose (mg/dL)					
IER+MED	104.3 ± 4.2	102.2 ± 4.2	0.374	−2.1 ± 2.4	0.928
DASH	104.9 ± 4.1	102.5 ± 4.1	0.294	−2.4 ± 2.3	
Aspartame transaminase (U/L)					
IER+MED	23.8 ± 1.8	18.1 ± 1.8	0.012	−5.7 ± 2.2	0.179
DASH	18.9 ± 1.7	17.3 ± 1.7	0.462	−1.6 ± 2.1	
Insulin (mU/L)					
IER+MED	13.9 ± 1.7	8.8 ± 1.7	<0.001	−5.1 ± 1.2	0.134
DASH	14.6 ± 1.6	12.0 ± 1.6	0.027	−2.5 ± 1.7	
Systolic blood pressure (mm Hg)					
IER+MED	133.2 ± 2.5	124.3 ± 2.7	<0.001	−9.0 ± 2.5	0.345
DASH	133.4 ± 2.5	127.7 ± 2.6	0.024	−5.7 ± 2.4	
Diastolic blood pressure (mm Hg)					
IER+MED	84.2 ± 1.7	77.5 ± 1.8	<0.001	−6.7 ± 1.5	0.124
DASH	86.2 ± 1.7	82.8 ± 1.8	0.021	−3.4 ± 1.4	

Data are presented as mean ± standard error of the mean (SEM). IER+MED: Intermittent energy restriction combined with a Mediterranean diet. DASH: Dietary Approaches to Stop Hypertension diet. ^1^ All data analyzed using an intention-to-treat approach with a linear mixed model for all 60 participants. ^2^ Within group difference from baseline to Week 12. ^3^ Between group difference from baseline to Week 12.

**Table 5:** Self-reported data collected during the 6-month post-intervention telephone interview for participants in the IER+MED group (*n* = 22) and the DASH group (*n* = 26), who completed the intervention.

Variable	IER+MED	DASH
Weight (kg)	75.5 ± 2.7	77.9 ± 3.2
Change in weight between Week 12 ^1^ and 6-month post-intervention (kg) ^2^	1.0 ± 3.8	1.1 ± 2.1 *
Physical activity (hours/week)	3.8 ± 0.4	3.6 ± 0.6
Able to follow the diet prescription for longer than 3 months (%)		
No	28.6	12.0
Yes	71.4	88.0
If yes, for how many more months?	5.0 ± 0.6	3.6 ± 0.5
Still following the diet prescription (%)		
No	33.3	56.0
Yes	66.7	44.0
If yes, following the diet the same, better, not as well, or other (%)		
Same	6.7	25.0
Better	0.0	0.0
Not as well	80.0	66.7
Same and not as well	13.3	0.0
Other	0.0	8.3
Would nutrition classes and/or food preparation classes been helpful (%)		
No	13.6	24.0
Yes	86.4	76.0

Data are presented as mean ± standard deviation (SD) or number (%). IER+MED: Intermittent energy restriction combined with a Mediterranean diet. DASH: Dietary Approaches to Stop Hypertension diet. ^1^ Weight measured at Week 12 visit. ^2^ Analyses completed using paired samples t-tests. * *p* ≤ 0.03.

## Supplementary Materials

The following are available online at https://www.mdpi.com/2072-6643/11/6/1386/s1, Table S1: Energy and macronutrient intake among participants completing the study, assessed using 4-day mobile food records (mFR™) captured by participants in the IER+MED group and DASH group over three time points, Table S2: Alcohol and micronutrient intake among participants completing the study, assessed using 4-day mobile food records (mFR™) captured by participants in the IER+MED group and DASH group over three time points, Table S3: Proportion of participants meeting US EAR for vitamin and mineral intake, assessed using 4-day mobile food records (mFR™) captured by participants in the IER+MED group and DASH group over three time points, Table S4: Baseline, Week 12, and change in body measures within and between trial groups, among participants completing the IER+MED (*n* = 26) or DASH diet (*n* = 28).
