# Supplementary material for: Effects of Intermittent Energy Restriction Combined with a Mediterranean Diet on Reducing Visceral Adiposity: A Randomized Active Comparator Pilot Study
Source: Nutrients. 2019 Jun 20;11(6):1386. doi: 10.3390/nu11061386 (PMC6627434; doi:10.3390/nu11061386)
Supplement: Supplementary file 1 [file nutrients-11-01386-s001.pdf]

Table S1. Energy and macronutrient intake among participants completing the study, assessed using 4-day mobile food records (mFR™) captured by participants in the IER+MED group and DASH group over three time points [1].

	Baseline	Weeks 5-6	*p* [2]	Week 11	*p* [3]
**Energy (kcal)**					
IER+MED [4]	1590 ± 78	1167 ± 81	<0.001	1085 ± 65	<0.001
IER [5]		960 ± 81		929 ± 62	
MED [6]		1222 ± 93		1144 ± 80	
DASH	1831 ± 115	1479 ± 91	0.004	1578 ± 93	0.003
**Protein (g)**					
IER+MED	72.9 ± 3.7	75.3 ± 5.5	0.668	72.6 ± 4.6	0.964
IER		70.6 ± 6.0		70.7 ± 5.3	
MED		75.0 ± 6.0		71.5 ± 5.2	
DASH	77.1 ± 4.8	69.3 ± 4.5	0.144	74.1 ± 4.3	0.380
**Protein (% energy)**					
IER+MED	18.7 ± 0.7	25.8 ± 1.0	<0.001	26.8 ± 1.1	<0.001
IER		29.5 ± 1.7		30.2 ± 1.4	
MED		24.7 ± 1.1		25.4 ± 1.2	
DASH	17.2 ± 0.7	18.9 ± 0.5	0.060	19.1 ± 0.5	0.002
**Carbohydrate (g)**					
IER+MED	182 ± 12	107 ± 8	<0.001	92 ± 7	<0.001
IER		70 ± 8		63 ± 4	
MED		119 ± 10		103 ± 10	
DASH	201 ± 13	168 ± 12	0.031	177 ± 12	0.023
**Carbohydrate (% energy)**					
IER+MED	45.1 ± 1.5	36.4 ± 1.5	<0.001	33.9 ± 1.5	<0.001
IER		28.7 ± 1.6		28.3 ± 1.8	
MED		39.2 ± 1.7		35.2 ± 2.0	
DASH	44.4 ± 1.3	44.8 ± 1.2	0.747	45.3 ± 1.4	0.497
**Total fat (g)**					
IER+MED	64 ± 4	51 ± 4	0.012	49 ± 3	0.001
IER		46 ± 5		45 ± 4	
MED		51 ± 5		52 ± 4	
DASH	79 ± 6	60 ± 4	0.001	66 ± 5	0.005
**Total fat (% energy)**					
IER+MED	36.4 ± 1.2	39.2 ± 1.5	0.150	40.4 ± 1.3	0.015
IER		43.4 ± 2.3		42.5 ± 1.7	
MED		37.4 ± 1.5		40.9 ± 2.1	
DASH	38.2 ± 1.2	36.4 ± 1.1	0.181	36.5 ± 1.3	0.232
**Saturated fatty acids (% energy)**					
IER+MED	11.6 ± 0.5	11.1± 0.6	0.563	11.7 ± 0.6	0.830
IER		11.2± 0.7		11.4 ± 0.6	
MED		10.9 ± 0.7		11.6 ± 0.8	
DASH	11.6 ± 0.5	10.8 ± 0.4	0.103	11.8 ± 0.5	0.789
**Monounsaturated fatty acids (% energy)**					
IER+MED	13.5 ± 0.4	15.0 ± 0.7	0.065	16.0 ± 0.7	0.001
IER		16.4 ± 1.0		17.2 ± 0.9	
MED		14.3 ± 0.7		16.9 ± 1.5	
DASH	14.4 ± 0.5	13.3 ± 0.5	0.081	13.7 ± 0.6	0.289
**Polyunsaturated fatty acids (% energy)**					
IER+MED	8.2 ± 0.6	9.5 ± 0.6	0.104	8.7 ± 0.5	0.470
IER		12.0 ± 1.3		9.8 ± 0.8	
MED		8.8 ± 0.6		8.5 ± 0.7	
DASH	9.1 ± 0.6	9.2 ± 0.6	0.859	7.8 ± 0.5	0.120
**Dietary fiber (g)**					
IER+MED	13.0 ± 1.2	12.7 ± 1.0	0.799	11.7 ± 1.0	0.256
IER		10.5 ± 0.9		9.3 ± 0.8	
MED		13.3 ± 1.1		12.8 ± 1.2	
DASH	13.5 ± 1.1	14.8 ± 1.3	0.420	14.3 ± 0.9	0.473

Data are presented as mean/day ± standard error of the mean (SEM). IER+MED: Intermittent energy restriction combined with a Mediterranean diet. DASH: Dietary Approaches to Stop Hypertension diet. [1] All data analyzed using a repeated measures mixed model for only the 54 participants who completed the study. [2] Within group difference from baseline to Weeks 5-6. [3] Within group difference from baseline to Week 11. [4] Weighted for five Mediterranean diet (MED) days and two intermittent energy restriction (IER) days. [5] IER days assessed using two days of 4-day mFR™. [6] MED days assessed using two days of 4-day mFR™.

Table S2. Alcohol and micronutrient intake among participants completing the study, assessed using 4-day mobile food records (mFR™) captured by participants in the IER+MED group and DASH group over three time points [1].

	Baseline	Weeks 5-6	Week 11
**Alcohol (g)**			
IER+MED [2]	1.5 ± 0.9	0.4 ± 0.3	0.3 ± 0.2
IER [3]		0.4 ± 0.3	0.1 ± 0.0
MED [4]		0.3 ± 0.3	0.3 ± 0.3
DASH	2.9 ± 1.4	2.1 ± 1.5	0.6 ± 0.4
**Total retinol (µg)**			
IER+MED	232 ± 17	209 ± 23	161 ± 21
IER		164 ± 18	140 ± 14
MED		219 ± 25	170 ± 22
DASH	261 ± 27	267 ± 41	304 ± 36
**Alpha-tocopherol (mg)**			
IER+MED	6 ± 0	6 ± 0	6 ± 1
IER		6 ± 0	6 ± 0
MED		6 ± 0	6 ± 1
DASH	8 ± 1	6 ± 1	7 ± 1
**Total alpha carotene (µg)**			
IER+MED	367 ± 89	586 ± 131	268 ± 63
IER		679 ± 188	332 ± 88
MED		528 ± 140	244 ± 79
DASH	369 ± 82	484 ± 91	486 ± 116
**Vitamin C (mg)**			
IER+MED	62 ± 9	81 ± 9	74 ± 8
IER		81 ± 9	72 ± 8
MED		79 ± 9	76 ± 9
DASH	60 ± 7	60 ± 6	58 ± 5
**Thiamin (mg)**			
IER+MED	1.3 ± 0.1	1.0 ± 0.1	0.9 ± 0.1
IER		0.8 ± 0.1	0.7 ± 0.1
MED		1.0 ± 0.1	0.9 ± 0.1
DASH	1.3 ± 0.1	1.1 ± 0.1	1.3 ± 0.1
**Riboflavin (mg)**			
IER+MED	1.5 ± 0.1	1.5 ± 0.1	1.4 ± 0.1
IER		1.3 ± 0.1	1.3 ± 0.1
MED		1.5 ± 0.1	1.4 ± 0.1
DASH	1.7 ± 0.2	1.5 ± 0.2	1.7 ± 0.1
**Niacin (mg)**			
IER+MED	19 ± 1	18 ± 1	17 ± 1
IER		16 ± 1	15 ± 1
MED		18 ± 1	17 ± 1
DASH	22 ± 2	17 ± 1	19 ± 1
**Vitamin B6 (mg)**			
IER+MED	1.5 ± 0.1	1.6 ± 0.1	1.5 ± 0.1
IER		1.4 ± 0.1	1.2 ± 0.1
MED		1.6 ± 0.1	1.5 ± 0.1
DASH	1.7 ± 0.2	1.5 ± 0.2	1.6 ± 0.1
**Folate (µg)**			
IER+MED	393 ± 27	306 ± 27	287 ± 26
IER		273 ± 22	225 ± 14
MED		310 ± 30	309 ± 27
DASH	355 ± 25	350 ± 39	428 ± 40
**Vitamin B12 (µg)**			
IER+MED	7.0 ± 1.2	5.7 ± 1.4	6.8 ± 2.5
IER		5.6 ± 1.5	5.4 ± 1.4
MED		5.5 ± 1.3	7.4 ± 2.2
DASH	5.6 ± 0.6	4.1 ± 0.5	4.9 ± 0.7

**Calcium (mg)**			
IER+MED	550 ± 36	550 ± 43	517 ± 41
IER		535 ± 41	481 ± 34
MED		536 ± 39	527 ± 40
DASH	586 ± 41	610 ± 67	661 ± 53
**Iron (mg)**			
IER+MED	12.9 ± 0.9	10.4 ± 0.7	9.7 ± 1.1
IER		9.2 ± 0.7	7.6 ± 0.6
MED		10.5 ± 0.8	10.6 ± 1.0
DASH	12.5 ± 0.9	10.8 ± 1.1	12.2 ± 1.0
**Magnesium (mg)**			
IER+MED	240 ± 16	233 ± 20	197 ± 15
IER		210 ± 17	165 ± 10
MED		236 ± 17	215 ± 14
DASH	254 ± 18	233 ± 20	259 ± 18
**Phosphorus (mg)**			
IER+MED	995 ± 46	1002 ± 72	943 ± 61
IER		912 ± 59	860 ± 49
MED		1006 ± 67	974 ± 58
DASH	1095 ± 69	994 ± 84	1099 ± 71
**Selenium (μg)**			
IER+MED	107 ± 6	97 ± 9	90 ± 7
IER		83 ± 7	87 ± 7
MED		99 ± 9	91 ± 7
DASH	111 ± 7	94 ± 7	102 ± 5
**Zinc (mg)**			
IER+MED	9.8 ± 0.6	8.5 ± 0.6	8.3 ± 0.5
IER		7.1 ± 0.5	6.9 ± 0.4
MED		8.8 ± 0.6	8.9 ± 0.6
DASH	10.3 ± 0.8	8.8 ± 0.8	9.9 ± 0.8

Data are presented as mean/day ± standard error of the mean (SEM). IER+MED: Intermittent energy restriction combined with a Mediterranean diet. DASH: Dietary Approaches to Stop Hypertension diet. [1]Analyzed using data from participants at baseline (IER+MED, *n*=30; DASH, *n*=30), and participants remaining in the study at Weeks 5-6 (IER+MED, *n*=26; DASH, *n*=30), and Week 11 (IER+MED, *n*=26; DASH, *n*=28). [2] Weighted for five Mediterranean diet (MED) days and two intermittent energy restriction (IER) days. [3] IER days assessed using two days of 4-day mFR. [4] MED days assessed using two days of 4-day mFR.

Table S3. Proportion of participants meeting US EAR for vitamin and mineral intake, assessed using 4-day mobile food records (mFR™) captured by participants in the IER+MED group and DASH group over three time points [1].

	Baseline	Weeks 5-6	Week 11
Calcium (mg)			
IER+MED [2]	5 (16.7)	2 (8.0)	2 (8.0)
DASH	8 (26.7)	10 (33.3)	10 (35.7)
Vitamin C (mg)			
IER+MED	9 (30.0)	13 (52.0)	13 (52.0)
DASH	8 (26.7)	13 (43.3)	12 (42.9)
Alpha-tocopherol (mg)			
IER+MED	2 (6.7)	0 (0.0)	1 (4.0)
DASH	3 (10.0)	1 (3.3)	2 (7.1)
Thiamin (mg)			
IER+MED	25 (83.3)	15 (60.0)	10 (40.0)
DASH	24 (80.0)	16 (53.3)	26 (92.9)
Riboflavin (mg)			
IER+MED	27 (90.0)	22 (91.7)	21 (84.0)
DASH	29 (96.7)	21 (72.4)	27 (96.4)
Niacin (mg)			
IER+MED	26 (86.7)	22 (88.0)	22 (88.0)
DASH	28 (93.3)	25 (83.3)	26 (92.9)
Vitamin B6 (mg)			
IER+MED	20 (66.7)	17 (68.0)	16 (64.0)
DASH	25 (83.3)	19 (63.3)	23 (82.1)
Folate (μg)			
IER+MED	21 (70.0)	11 (44.0)	9 (36.0)
DASH	16 (53.3)	12 (40.0)	19 (67.9)
Vitamin B12 (μg)			
IER+MED	29 (96.7)	22 (88.0)	23 (92.0)
DASH	29 (96.7)	24 (80.0)	24 (85.7)
Iron (mg)			
IER+MED	27 (90.0)	22 (88.0)	20 (80.0)
DASH	27 (90.0)	21 (70.0)	25 (89.3)
Magnesium (mg)			
IER+MED	9 (30.0)	5 (20.0)	5 (20.0)
DASH	5 (16.7)	10 (33.3)	11 (39.3)
Phosphorus (mg)			
IER+MED	29 (96.7)	23 (92.0)	21 (84.0)
DASH	30 (100.0)	24 (80.0)	26 (92.9)
Selenium (μg)			
IER+MED	29 (96.7)	23 (92.0)	22 (88.0)
DASH	30 (100.0)	27 (90.0)	28 (100.0)
Zinc (mg)			
IER+MED	21 (70.0)	16 (64.0)	14 (56.0)
DASH	23 (76.7)	17 (56.7)	22 (78.6)

Data are presented as number (%). IER+MED: Intermittent energy restriction combined with a Mediterranean diet. DASH: Dietary Approaches to Stop Hypertension diet.[1] Analyzed using data from participants in the study at baseline (IER+MED, *n*=30; DASH, *n*=30), and participants remaining in the study at Weeks 5-6 (IER+MED, *n*=26; DASH, *n*=30), and Week 11 (IER+MED, *n*=26; DASH, *n*=28). [2] Weighted for five Mediterranean diet (MED) days and two intermittent energy restriction (IER) days.

Table S4. Baseline, Week 12, and change in body measures within and between trial groups, among participants completing the IER+MED (n = 26) or DASH diet (n = 28) [1].

	**Baseline**	**Week 12**	p [2]	**Change**	p [3]
Weight (kg)					
IER+MED	81.0 ± 2.4	75.1 ± 2.4	<0.001	-5.9 ± 0.7	0.005
DASH	80.4 ± 2.3	77.2 ± 2.3	<0.001	-3.2 ± 0.6	
Body mass index (kg/m²)					
IER+MED	30.8 ± 0.6	28.6 ± 0.6	<0.001	-2.2 ± 0.2	0.001
DASH	30.6 ± 0.6	29.4 ± 0.6	<0.001	-1.2 ± 0.2	
Waist circumference (cm)					
IER+MED	101.0 ± 1.7	94.0 ± 1.7	<0.001	-7.0 ± 0.8	0.020
DASH	100.2 ± 1.6	95.7 ± 1.6	<0.001	- 4.5 ± 0.7	
Hip circumference (cm)					
IER+MED	107.9 ± 1.4	102.7 ± 1.4	<0.001	-5.3 ± 0.5	0.019
DASH	107.0 ± 1.4	103.6 ± 1.4	<0.001	-3.4 ± 0.5	
Body fat (%)					
IER+MED	32.9 ± 1.3	30.9 ± 1.3	<0.001	-2.0 ± 0.4	0.023
DASH	32.9 ± 1.2	32.1 ± 1.2	0.024	-0.8 ± 0.4	
Fat mass (kg)					
IER+MED	26.6 ± 1.2	23.3 ± 1.2	<0.001	-3.3 ± 0.4	0.004
DASH	26.2 ± 1.2	24.6 ± 1.2	<0.001	-1.6 ± 0.4	
Muscle mass (kg)					
IER+MED	22.4 ± 1.0	21.3 ± 1.0	<0.001	-1.1 ± 0.2	0.012
DASH	22.2 ± 1.0	21.7 ± 1.0	0.005	-0.5 ± 0.2	
Total lean body mass (kg)					
IER+MED	54.1 ± 2.0	51.8 ± 2.0	<0.001	-2.3 ± 0.4	0.041
DASH	53.9 ± 1.8	52.7 ± 1.9	0.002	-1.2 ± 0.4	
Visceral adipose tissue area (cm²)					
IER+MED	136.0 ± 6.2	113.2 ± 6.2	<0.001	-22.8 ± 3.6	0.014
DASH	130.1 ± 6.0	120.0 ± 6.0	0.006	-10.0 ± 3.5	
Subcutaneous adipose tissue area (cm²)					
IER+MED	375.5 ± 17.8	327.1 ± 17.8	<0.001	-48.4 ± 6.4	<0.001
DASH	355.2 ± 17.1	340.4 ± 17.1	0.020	-15.0 ± 6.1	
VAT/SAT ratio [6]					
IER+MED	0.38 ± 0.02	0.37 ± 0.02	0.146	-0.01 ± 0.01	0.892
DASH	0.38 ± 0.02	0.37 ± 0.02	0.090	-0.01 ± 0.01	

Data are presented as mean ± standard error of the mean (SEM). IER+MED: Intermittent energy restriction combined with a Mediterranean diet. DASH: Dietary Approaches to Stop Hypertension diet.[1] All data analyzed using a repeated measures mixed model for only the 54 participants who completed the study. [2] Within group difference from baseline to Week 12. [3] Between group difference (IER+MED vs. DASH) from baseline to Week 12. [6] Ratio of visceral adipose tissue area to subcutaneous adipose tissue area.

CONSORT 2010 checklist of information to include when reporting a pilot or feasibility trial*

Section/Topic	Item No	Checklist item	Reported on page No
**Title and abstract**			
	1a	Identification as a pilot or feasibility randomised trial in the title	1
	1b	Structured summary of pilot trial design, methods, results, and conclusions (for specific guidance see CONSORT abstract extension for pilot trials)	1
**Introduction**			
Background and objectives	2a	Scientific background and explanation of rationale for future definitive trial, and reasons for randomised pilot trial	1-2
	2b	Specific objectives or research questions for pilot trial	2
**Methods**			
Trial design	3a	Description of pilot trial design (such as parallel, factorial) including allocation ratio	2-5
	3b	Important changes to methods after pilot trial commencement (such as eligibility criteria), with reasons	N/A
Participants	4a	Eligibility criteria for participants	3
	4b	Settings and locations where the data were collected	2,3,6,7
	4c	How participants were identified and consented	3
Interventions	5	The interventions for each group with sufficient details to allow replication, including how and when they were actually administered	5,6
Outcomes	6a	Completely defined prespecified assessments or measurements to address each pilot trial objective specified in 2b, including how and when they were assessed	3,5,6,7
	6b	Any changes to pilot trial assessments or measurements after the pilot trial commenced, with reasons	N/A
	6c	If applicable, prespecified criteria used to judge whether, or how, to proceed with future definitive trial	N/A
Sample size	7a	Rationale for numbers in the pilot trial	3
	7b	When applicable, explanation of any interim analyses and stopping guidelines	7
Randomisation:			
Sequence generation	8a	Method used to generate the random allocation sequence	4
	8b	Type of randomisation(s); details of any restriction (such as blocking and block size)	4
Allocation concealment mechanism	9	Mechanism used to implement the random allocation sequence (such as sequentially numbered containers), describing any steps taken to conceal the sequence until interventions were assigned	4
Implementation	10	Who generated the random allocation sequence, who enrolled participants, and who assigned participants to interventions	4
Blinding	11a	If done, who was blinded after assignment to interventions (for example, participants, care providers, those assessing outcomes) and how	4
	11b	If relevant, description of the similarity of interventions	5,6,7
Statistical methods	12	Methods used to address each pilot trial objective whether qualitative or quantitative	7,8
**Results**			
Participant flow (a diagram is strongly recommended)	13a	For each group, the numbers of participants who were approached and/or assessed for eligibility, randomly assigned, received intended treatment, and were assessed for each objective	4
	13b	For each group, losses and exclusions after randomisation, together with reasons	4
Recruitment	14a	Dates defining the periods of recruitment and follow-up	2
	14b	Why the pilot trial ended or was stopped	N/A
Baseline data	15	A table showing baseline demographic and clinical characteristics for each group	8
Numbers analysed	16	For each objective, number of participants (denominator) included in each analysis. If relevant, these numbers should be by randomised group	8,10,12,13,14
Outcomes and estimation	17	For each objective, results including expressions of uncertainty (such as 95% confidence interval) for any estimates. If relevant, these results should be by randomised group	8,10,12,13,14
Ancillary analyses	18	Results of any other analyses performed that could be used to inform the future definitive trial	11
Harms	19	All important harms or unintended effects in each group (for specific guidance see CONSORT for harms)	8
	19a	If relevant, other important unintended consequences	N/A
**Discussion**			

Limitations	20	Pilot trial limitations, addressing sources of potential bias and remaining uncertainty about feasibility	16
Generalisability	21	Generalisability (applicability) of pilot trial methods and findings to future definitive trial and other studies	14,15
Interpretation	22	Interpretation consistent with pilot trial objectives and findings, balancing potential benefits and harms, and considering other relevant evidence	14,15
	22a	Implications for progression from pilot to future definitive trial, including any proposed amendments	15,16
**Other information**			
Registration	23	Registration number for pilot trial and name of trial registry	1
Protocol	24	Where the pilot trial protocol can be accessed, if available	1
Funding	25	Sources of funding and other support (such as supply of drugs), role of funders	17
	26	Ethical approval or approval by research review committee, confirmed with reference number	3

Citation: Eldridge SM, Chan CL, Campbell MJ, Bond CM, Hopewell S, Thabane L, et al. CONSORT 2010 statement: extension to randomised pilot and feasibility trials. BMJ. 2016;355.

*We strongly recommend reading this statement in conjunction with the CONSORT 2010, extension to randomised pilot and feasibility trials, Explanation and Elaboration for important clarifications on all the items. If relevant, we also recommend reading CONSORT extensions for cluster randomised trials, non-inferiority and equivalence trials, non-pharmacological treatments, herbal interventions, and pragmatic trials. Additional extensions are forthcoming: for those and for up to date references relevant to this checklist, see www.consort-statement.org.
